# Metabolomics-based discrimination of patients with remitted depression from healthy controls using ^1^H-NMR spectroscopy

**DOI:** 10.1038/s41598-021-95221-1

**Published:** 2021-08-02

**Authors:** Ching-I. Hung, Gigin Lin, Meng-Han Chiang, Chih-Yung Chiu

**Affiliations:** 1 Department of Psychiatry, Chang-Gung Memorial Hospital at Linkou, Taoyuan, Taiwan, ROC; 2 College of Medicine, Chang Gung University, Taoyuan, Taiwan, ROC; 3 Department of Medical Imaging and Intervention, Imaging Core Laboratory, Institute for Radiological Research, Chang Gung Memorial Hospital at Linkou, Taoyuan, Taiwan, ROC; 4 Clinical Metabolomics Core Laboratory, Chang Gung Memorial Hospital at Linkou, Taoyuan, Taiwan, ROC; 5 Division of Pediatric Pulmonology, Department of Pediatrics, Chang Gung Memorial Hospital at Linkou, 5 Fu-Shing St., Kweishan, Taoyuan, 333 Taiwan, ROC

**Keywords:** Neuroscience, Biomarkers, Diseases, Medical research

## Abstract

The aim of the study was to investigate differences in metabolic profiles between patients with major depressive disorder (MDD) with full remission (FR) and healthy controls (HCs). A total of 119 age-matched MDD patients with FR (*n* = 47) and HCs (*n* = 72) were enrolled and randomly split into training and testing sets. A ^1^H-nuclear magnetic resonance (NMR) spectroscopy-based metabolomics approach was used to identify differences in expressions of plasma metabolite biomarkers. Eight metabolites, including histidine, succinic acid, proline, acetic acid, creatine, glutamine, glycine, and pyruvic acid, were significantly differentially-expressed in the MDD patients with FR in comparison with the HCs. Metabolic pathway analysis revealed that pyruvate metabolism via the tricarboxylic acid cycle linked to amino acid metabolism was significantly associated with the MDD patients with FR. An algorithm based on these metabolites employing a linear support vector machine differentiated the MDD patients with FR from the HCs with a predictive accuracy, sensitivity, and specificity of nearly 0.85. A metabolomics-based approach could effectively differentiate MDD patients with FR from HCs. Metabolomic signatures might exist long-term in MDD patients, with metabolic impacts on physical health even in patients with FR.

## Introduction

Major depressive disorder (MDD) is a common mental disorder. However, no robust objective laboratory test is available for the diagnosis of MDD or evaluation of the severity of depression. A metabolomics-based approach can be employed to identify products of a given biochemical system and metabolic substrates, and therefore this approach has emerged as a method by which to increase our understanding of diseases and biological systems in a large-scale manner^1^. The technology of metabolomics offers significant potential as a tool to investigate the diagnosis of diseases and responses to medications. Metabolomics has been used in MDD-related research, such as to evaluate the severity of depression^2^, identify biomarkers of MDD^3–9^, for predictive diagnosis of MDD^10–13^, identify metabolic profiles post-antidepressant treatment^14–17^, pinpoint biomarkers of metabolites for drug response phenotypes^18,19^, and differentiate MDD from bipolar disorder^12,20,21^.

Full remission (FR) of depression is a treatment goal for patients with MDD. One of the commonly-used definitions of FR is a 17-item Hamilton Depression Rating Scale (HAMD) score ≤ 7^22^. However, MDD with FR does not equate to achieving health^23,24^. For example, cognitive dysfunction, which may hinder functional recovery, is one of the common residual symptoms of depression, and may persist during the remission phase^25^. This raises two interesting questions: (1) are there any differences in the metabolic profiles between MDD patients with FR and healthy controls (HCs), and (2) is it possible to establish an algorithm based on metabolites as biomarkers to differentiate MDD patients with FR from HCs?

The majority of studies of metabolomics in MDD patients, as described above, have been concerned with identifying biomarkers or obtaining a predictive diagnosis of MDD, or predicting the response to antidepressants. Few studies have focused on investigating differences in metabolite expressions between MDD patients with FR and HCs using targeted metabolomics analysis^26,27^, and to our knowledge, no study has comprehensively investigated differences in metabolite levels in peripheral plasma between MDD patients with FR and HCs. An algorithm based on metabolomics analysis to differentiate MDD patients with FR from HCs is still lacking. However, investigation of the above two issues is important, because MDD has negative impacts on multiple physical systems^28–30^. Abnormalities in metabolites among MDD patients with FR might be associated with long-term negative impacts on physical health. Furthermore, recurrence is common in MDD, and investigation of these issues may provide clues as to the recurrence of depression and subsequent prevention of depression.

Therefore, this study aimed to comprehensively investigate the differences in metabolomic profiles in peripheral plasma between patients with MDD with FR and HCs, and to then establish an algorithm based on metabolomics analysis to differentiate MDD patients with FR from HCs. We hypothesized that an algorithm based on metabolomics analysis could be effective in differentiating MDD patients with FR from HCs.

## Methods

### Subjects

The subjects included in this study were nested within a project that examined MDD patients and were recruited at the 10-year follow-up point from August 2014 to December 2016^29,31,32^. At baseline (from January 2004 to August 2007), patients diagnosed with MDD in that project were enrolled from outpatient clinics of the Psychiatric Department of Chang Gung Memorial Hospital at Linkou, a medical center in northern Taiwan. The outpatients fulfilled the criteria for MDD, and were diagnosed using the Structured Clinical Interview for DSM-IV-text revision (TR) Axis I Disorders^33^.

At baseline, 229 participants with MDD were enrolled, then were treated by antidepressants. At the 10-year follow-up point, 137 (47.2%) subjects attended follow-up. The severity of depression was evaluated using the 17-item Hamilton Depression Rating Scale (HAMD)^34^ administered by a psychiatrist, and among the 137 subjects, a total of 47 MDD patients were in FR, which was defined as a HAMD score ≤ 7^22^, and had been medication-free for at least 6 months and had no history of substance abuse or dependence.

Sixty-seven healthy persons were simultaneously enrolled as controls. The exclusion criteria for the HCs were as follows: (1) any current or previous lifetime history of neurological or DSM-IV-TR axis I/II diagnoses; (2) systemic medical diseases, such as hypertension, diabetes mellitus, and others; and (3) any family history of psychiatric disorders. The project was approved by the Institutional Review Board of Chang Gung Memorial Hospital (No. 105-5895C). Based on the guidelines regulated in the Declaration of Helsinki, written informed consent was acquired from all subjects.

The enrolled subjects, including 47 MDD patients with FR and 67 HCs, were then randomly split (3/5 for training, 2/5 for testing) into training (30 MDD patients with FR and 42 HCs) and testing sets (17 MDD patients with FR and 30 HCs) for algorithm development. Twelve-hour fasting plasma samples of the 47 MDD patients with FR and 67 HCs were collected and analyzed at the 10-year follow-up point. Fasting plasma parameters including glucose, cholesterol, or triglyceride, alanine aminotransferase (ALT), and aspartate aminotransferase (AST) analyzed by completely automated methods at clinical laboratories were also analyzed.

### Plasma sample preparation

Fasting blood samples for plasma collection were obtained at 9–10 a.m., aliquoted and stored immediately at − 80 °C until analysis. Thawed plasma samples were centrifuged at 12,000×*g* at 4 °C for 30 min. 500 μL of plasma supernatant were mixed with 500 μL of 0.075 M phosphate buffer (pH 7.40) in 20% deuterium water containing 0.08% 3-(trimethylsilyl)-propionic-2,2,3,3-d_4_ acid sodium salt (TSP) as an internal chemical shift reference standard. The mixed samples were vortexed for 20 s and centrifuged at 12,000×*g* at 4 °C for 30 min, following which 600 μL of the supernatant were loaded into a standard 5-mm NMR tube (Bruker BioSpin, Billerica, MA, USA) for further analysis.

### Nuclear magnetic resonance (NMR) spectrum acquisition

NMR experiments were performed at Chang Gung Healthy Aging Research Center, Taiwan. ^1^H-NMR spectra were acquired on a Bruker Avance 600-MHz spectrometer (Bruker-Biospin GmbH, Karlsruhe, Germany) equipped with a 5-mm CPTCI ^1^H cryoprobe. Temperature was controlled at 300 K throughout the experiments. Relaxation-edited spectra were acquired using Carr–Purcell–Meiboom–Gill (CPMG)-presat pulse sequence. In CPMG method, a series of 180° pulse was applied with the radio-frequency (RF) pulses in 27.24 μs and the water presaturation bandwidth 25 Hz. Low-power water pre-saturation pulse sequence was used for water signal suppression during the relaxation time of 4 s. For each spectrum, 64 transients were collected into 64 K data points using a spectral window of 20 ppm during a relaxation time of 4 s. The temperature-controlled Bruker SampleJet automation unit was installed for sample handling and laboratory automation. Prior to Fourier transformation, all ^1^H-NMR spectra were processed with zero-filling and exponential line-broadening of 0.3 Hz. The acquired spectra were manually phased, baseline corrected, and the internal TSP signal calibrated to δ 0.0 ppm using TopSpin 3.2 software (Bruker BioSpin, Rheinstetten, Germany).

### NMR data processing and analysis

NMRProcFlow (https://www.nmrprocflow.org), an open-source software, provides comprehensive tools for the processing and visualization of 1D NMR data. The raw ^1^H-NMR spectra were imported into NMRProcFlow 1.3 for ppm calibration, baseline correction, alignment, spectra bucketing and data normalization^35^. Spectra bucketing was performed using the method of intelligent bucketing and variable size bucketing with the full range of 10.0–0.00 ppm^36^. Metabolite identification was performed using Chenomx NMR Suite 8.0 professional software (Chenomx Inc., Edmonton, AB, Canada). The compounds were identified by comparing spectra to database Chenomx 600 MHz Version 9 (Chenomx Inc., Edmonton, Canada) with 332 metabolites in this particular database. A standard two-dimensional (2D) NMR experiment (^1^H and ^13^C NMR spectrum) was conducted on a pooled plasma sample and metabolites were further assigned by comparison with reference spectra from the Human Metabolome Database (HMDB). The area of individual resonances of glucose metabolite was significantly correlated with biochemical glucose concentration (Supplementary Fig. S1). The exported bucketing data of the ^1^H-NMR spectra were uploaded to MetaboAnalyst 4.0 (http://www.metaboanalyst.ca) with mean-centered, generalized log transformation and scaled by Pareto scaling. To identify metabolites that may be used to distinguish MDD patients with FR from healthy controls, partial least squares-discriminant analysis (PLS-DA) was applied with the variable importance in projection (VIP) score and fold-change values. Pathway analysis of the potential metabolites selected owing to a *p*-value lower than 0.05 was carried out to identify the implicated pathways. The potential metabolites were selected from the training samples, and Receiver Operating Characteristic (ROC) analysis was performed to investigate the accuracy of the training and testing models using four well-established algorithms, including PLS-DA, Random Forest, Support Vector Machine (SVM) and Logistic Regression Models. One-hundred cross-validations were performed to obtain a more reliable prediction model and the permutation test was used 1000 times to evaluate the performance of the model.

## Results

### Subjects

Table 1 shows the differences in demographic variables and biochemical indices between the MDD patients with FR and the HCs in the training and testing groups. There were no significant differences in age, gender, BMI, fasting plasma glucose, cholesterol, or triglyceride levels between groups. A significant difference was noted in the HAMD score between the MDD patients with FR and the HCs in the training group; however, both scores were within the range of FR (HAMD score ≤ 7).

**Table 1:** Demographic variables and biochemical indices in the MDD patients with full remission and the healthy controls.

	Training group		Testing group	
	MDD with remission	Healthy controls	MDD with remission	Healthy controls
Number	30	42	17	30
Age (years)	42.1 ± 9.2	41.2 ± 7.3	38.2 ± 5.2	40.6 ± 8.7
Female (%)	70.0	71.4	58.8	66.7
HAMD score	3.7 ± 2.0	1.5 ± 2.4*	2.9 ± 1.9	2.1 ± 4.2
BMI	24.2 ± 4.8	22.8 ± 3.7	22.8 ± 4.3	23.8 ± 3.5
Fasting plasma glucose (mg/dL)	92.6 ± 19.7	89.5 ± 10.2	104.9 ± 62.2	86.7 ± 5.7
Cholesterol	190.8 ± 29.2	185.5 ± 25.1	193.5 ± 24.2	191.5 ± 30.8
Triglycerides (mg/dL)	104.9 ± 61.1	94.4 ± 61.1	97.4 ± 60.8	94.5 ± 51.3
AST	23.4 ± 9.7	22.6 ± 5.4	22.9 ± 4.9	25.0 ± 7.4
ALT	21.8 ± 15.8	19.1 ± 12.1	19.2 ± 9.7	23.7 ± 16.4

Full remission was defined as a HAMD score ≤ 7.

*MDD* major depressive disorder, *HAMD* Hamilton Depression Rating Scale, *BMI* body mass index, *ALT* alanine aminotransferase, *AST* aspartate aminotransferase.

*p < 0.05.

### Metabolites significantly differentially-expressed between the MDD patients with FR and the HCs in the training group

**Figure 1:**
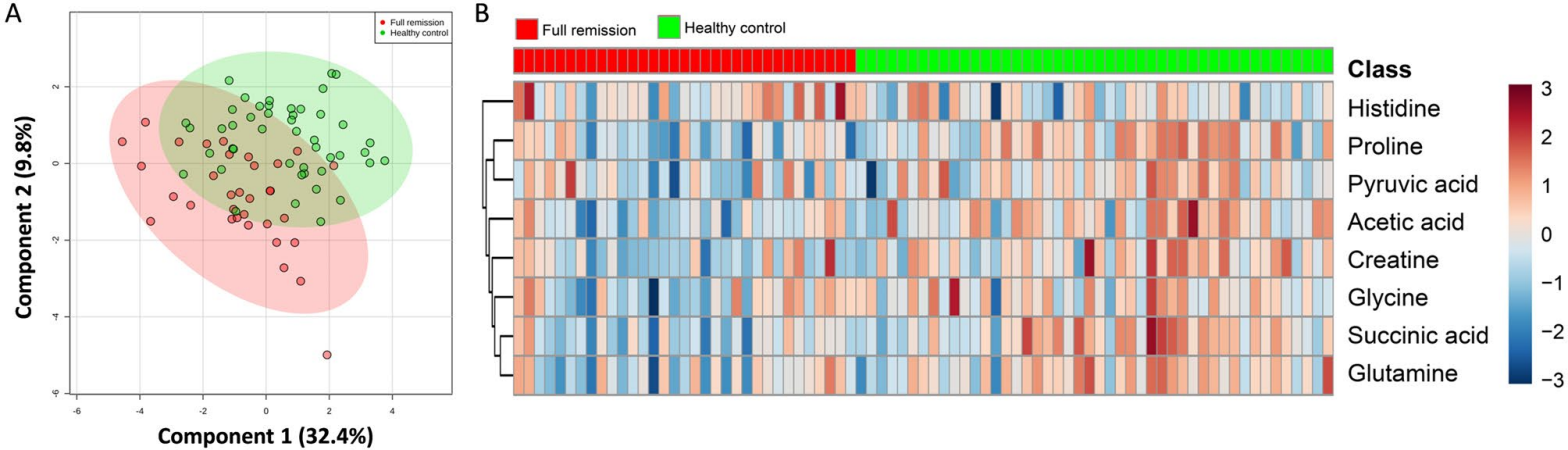
PLS-DA score plots from the analysis of ^1^H-NMR spectra using plasma samples and a heat map of eight metabolites significantly differentially-expressed between the major depressive disorder (MDD) patients with full remission (FR) and healthy controls (HCs). (**A**) Two-dimensional scatter plot showing the model’s degree of separation between the two groups: x axis, component 1 (% of total variance); y axis, component 2 (% of total variance). (**B**) Each column represents a plasma sample and each row represents the expression profile of a metabolite. The fold changes from the overall mean concentration are shown in a color-coded manner, with blue representing a decrease and red an increase.

**Figure 2:**
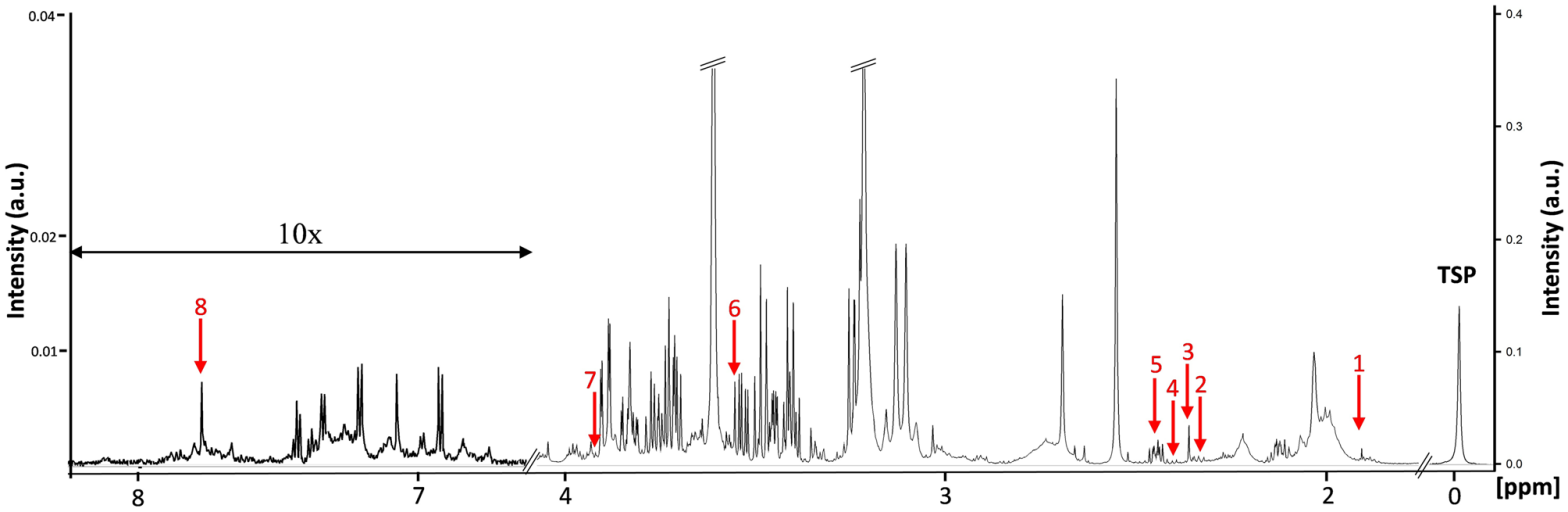
Representative 600 MHz ^1^H-NMR spectra of plasma showing the selected eight metabolite signals (δ1–9). x axis, parts per million (ppm); y axis, intensity (a.u.). 1, Acetic acid; 2, Proline; 3, Pyruvic acid; 4, Succinic acid; 5, Glutamine; 6, Glycine; 7, Creatine; 8, Histidine.

^1^H-NMR spectra obtained from plasma corresponded to 27 known metabolites (Supplementary Table S1). Metabolites that contributed to discrimination between the groups were identified using supervised PLS-DA (Fig. 1A, score plots). Table 2 shows the metabolites significantly differentially-expressed between the MDD patients with FR and the HCs in the training group. Compared with the HCs, eight metabolites were found to be significantly associated with the MDD patients with FR (*p* < 0.05), among which seven metabolites, including succinic acid, proline, acetic acid, creatine, glutamine, glycine, and pyruvic acid, had significantly lower expressions in the MDD patients with FR, while in contrast histidine had a significantly higher expression in the MDD patients with FR than in the HCs. Figure 1B shows a heatmap of these eight metabolites clustered using Hierarchical Clustering. A representative 600 MHz ^1^H-NMR spectra of selected eight metabolite signals are shown in Fig. 2.

**Table 2:** Significantly differentially-expressed metabolites between the MDD patients with full remission and the healthy controls.

Metabolite	Chemical shift (ppm)	VIP score	Fold change	*p*
Succinic acid	2.394–2.397 (s)	1.32	0.85	< 0.001
Proline	2.322–2.357 (m)	1.79	0.75	< 0.001
Acetic acid	1.907–1.914 (s)	1.37	0.83	< 0.001
Creatine	3.918–3.926 (s)	1.03	0.89	0.001
Glutamine	2.403–2.409 (m)	0.79	0.93	0.005
Glycine	3.548–3.565 (s)	0.63	0.94	0.020
Pyruvic acid	2.357–2.369 (s)	0.77	0.91	0.032
Histidine	7.760–7.783 (s)	1.39	1.06	0.039

*MDD* major depressive disorder, *VIP* variable importance in the projection, *s* singlet, *m* multiplet.

### Metabolic pathway associated with MDD patients with FR

Table 3 shows functional pathways of the metabolic network associated with the MDD patients with FR. Pyruvate metabolism via the tricarboxylic acid (TCA) cycle linked to amino acid metabolism, including alanine, aspartate and glutamate; arginine and proline; and glycine, serine and threonine metabolisms, was significantly associated with the MDD patients with FR (*p* < 0.01).

**Table 3:** Functional pathway analysis of metabolites associated with MDD with full remission.

Pathway name	Match status	Metabolites^a^	*P*	FDR	Impact
Alanine, aspartate and glutamate metabolism	3/24	Pyruvic acid, glutamine, succinic acid	< 0.001	0.002	0.207
Aminoacyl-tRNA biosynthesis	4/75	Histidine, glutamine, glycine, proline	< 0.001	0.002	0.000
Arginine and proline metabolism	4/77	Glutamine, proline, creatine, pyruvic acid	< 0.001	0.002	0.134
Nitrogen metabolism	3/39	Glutamine, histidine, glycine	< 0.001	0.004	0.000
Glycine, serine and threonine metabolism	3/48	Glycine, creatine, pyruvic acid	< 0.001	0.006	0.188
Taurine and hypotaurine metabolism	2/20	Pyruvic acid, acetic acid	0.002	0.020	0.022
Citrate cycle (TCA cycle)	2/20	Succinic acid, pyruvic acid	0.002	0.020	0.105
Glycolysis or Gluconeogenesis	2/31	Pyruvic acid, acetic acid	0.004	0.041	0.096
Pyruvate metabolism	2/32	Pyruvic acid, acetic acid	0.005	0.041	0.282

*MDD* major depressive disorder, *FDR* false discovery rate, *TCA* tricarboxylic acid.

^a^Metabolites for which *p* < 0.05 were selected.

### Model of metabolites in MDD patients with FR

**Figure 3:**
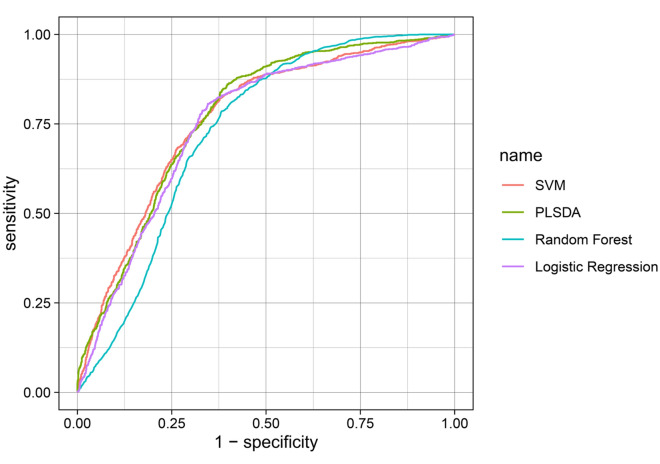
Receiver operating characteristics (ROC) curves for supportive vector machine (SVM), PLS-DA, random forest, and logistic regression models.

Table 4 shows the performance of the model of metabolites in terms of discriminating the MDD patients with FR from the HCs using four types of machine learning algorithm. Figure 3 shows the ROC curves for the SVM, PLS-DA, random forest, and logistic regression models. The model included the eight metabolites that had been identified as being significantly associated with the MDD patients with FR, with a highest AUC value of 0.784 and a highest predictive accuracy of 0.715 in the traing group (*P*_permutation test_ < 0.05). Using linear SVM classification in the testing group, the predictive accuracy, sensitivity, and specificity were 0.846, with a positive predictive value of 0.733 and a negative predictive value of 0.917.

**Table 4:** Model of metabolites in MDD with full remission using different types of machine learning algorithm.

Model metabolite^a^	Machine learning model	Training model				Testing model				
		AUC	*P*_permutation test_^b^	Predictive accuracy	*P*_permutation test_	Predictive accuracy	Sensitivity	Specificity	Positive predictive value	Negative predictive value
Succinic acid Proline Acetic acid Creatine Glutamine Glycine Pyruvic acid Histidine	Linear SVM	0.784	0.007	0.707	0.011	0.846	0.846	0.846	0.733	0.917
	PLS-DA	0.779	0.003	0.705	0.011	0.846	0.923	0.808	0.706	0.955
	Random FOREST	0.738	0.007	0.677	0.029	0.821	0.769	0.846	0.714	0.880
	Logistic regression	0.772	0.004	0.715	0.005	0.821	0.769	0.846	0.714	0.880

*MDD* major depressive disorder, *AUC* area under the receiver operating characteristic curve, *SVM* support vector machine, *PLS-DA* partial least squares-discriminant analysis.

^a^Metabolites for which *p* < 0.05 were selected.

^b^1000 random permutations were performed for validation testing.

## Discussion

This study demonstrated the potential of metabolic profiling in MDD patients with FR. A model based on metabolomics analysis using machine learning could effectively differentiate MDD patients with FR from HCs. Several studies have reported differences in metabolomes between MDD patients in a depressive episode and HCs^5,12,37^. Our findings implied the long-term existence of biological characteristics of MDD, even in patients with FR. Despite the fact that MDD with FR does not equate to achieving health in terms of clinical symptoms, such as cognitive dysfunction^23,25^, our results further demonstrated that FR of depression might not be equivalent to biological health based on the aspect of metabolomics.

In this study, eight metabolites were identified as being significantly differentially-expressed between the MDD patients with FR and the HCs. One review article identified several differentially-expressed metabolites between patients with MDD and controls from 46 studies^37^. Different studies of MDD patients might present controversial results in terms of elevated or decreased levels of metabolites as compared with HCs^37–39^. However, all eight metabolites identified in this study had been previously reported to be associated with MDD^37^, with the exception of acetic acid. Among these metabolites, significantly lower levels of l-glutamine and pyruvic acid were identified in the MDD patients with FR as compared with the HCs, which was compatible with the findings of an integrated meta-analysis of metabolites in MDD patients^37^. As was the case in this study, lower levels of succinic acid and glutamine have been reported to be significant in the diagnosis of MDD using metabolomics analysis^12^. However, in contrast to the decreased level of proline and elevated level of histidine observed in this study, antidepressant-free MDD patients have previously been reported to have a conversely increased level of proline and decreased level of l-histidine^37^.

A recent study reported that serum levels of methionine, phenylalanine, tryptophan, and tyrosine were significantly decreased in MDD patients compared to HCs^40^. Three of these four metabolites including methionine, tryptophan, and tyrosine related to aminoacyl-tRNA biosynthesis, glycine, serine and threonine metabolism, and citrate cycle were associated with MDD as in this study. Higher serum serine levels have reported to be significantly higher in patients with depression^41^. In addition, plasma levels of glutamate, glutamine, glycine, and taurine were found to be significantly increased in the depressed patients, particularly reflecting the severity of depression^42^. Despite the differences between studies, the available evidence suggests the importance of amino acid metabolites in patients with depressive disorder.

Amino acids, in particular glycine, glutamate, and glutamine, have been reported to significantly affect macrophage atherogenicity through modulation of the cellular triglyceride metabolism^43^. Most importantly, the anti-atherogenic properties of glycine have been further confirmed in vivo^43^. In this study, the MDD patients with FR appeared to have steady, low glycine levels, which may imply a risk of atherosclerosis in MDD patients, even with FR. Indeed, depression is clinically associated with an increased risk of cardiovascular diseases^28,30^. The findings of this study indicated that MDD patients might suffer persistent metabolic impacts on physical health, despite FR of the disease.

Nine functional metabolic pathways associated with MDD with FR were identified in this study, most of which have been reported previously^37^. Amino acid metabolism in the peripheral blood, such as nitrogen metabolism and aminoacyl-tRNA biosynthesis, appeared to be prominently associated with MDD patients^44,45^. The majority of the differentially-expressed metabolites identified in this study were significantly lower in the MDD patients with FR. In fact, previous studies have also reported some reductions in amino acid bioavailability in MDD patients^37,38^.

Several points are worthy of note. (1) Ali-Sisto et al. identified a significant difference between MDD patients and HCs in purine metabolism by analysis of fasting serum samples; however, there were no significant differences in metabolite levels between remitted and non-remitted MDD patients^26^. Most of the metabolites identified in this study had been reported in previous studies that investigated differences in metabolites between MDD patients in a depressive episode and HCs^37^. These results demonstrated that metabolomic signatures of MDD might not disappear, even with FR. (2) Kaddurah-Daouk et al. reported significant differences in tryptophan and tyrosine metabolism in cerebrospinal fluid in MDD patients with FR in comparison with HCs^27^. Our study ascertained that differences in metabolomics between MDD patients with FR and HCs were also present in peripheral plasma. (3) There is a possibility that people in the community who have similar metabolomic characteristics to MDD patients with FR may be at greater risk of the onset of depression; however, this hypothesis requires more evidence for confirmation.

There were some limitations and bias in this study. (1) The course of depression fluctuates, and it was difficult to clarify how long the patients with MDD had been in FR at the time point of the investigation. It was also unknown whether the duration of FR might affect the results of metabolomics analysis. (2) The HAMD score in the MDD patients with FR was still significantly higher than that in the HCs in the training group. It was unknown whether this difference in the HAMD score was a factor associated with metabolomic differences between the MDD patients with FR and the HCs. (3) The study did not control the phase of menstrual cycle, which might affect metabolomic profiles^46^, in female subjects. This might cause bias.

## Conclusion

There were significant differences in the expressions of eight metabolites between the MDD patients with FR and the HCs. Pyruvate metabolism via the TCA cycle linked to amino acid metabolism may play a biological role in the potential depression status. Using machine learning Linear SVM, a model containing the eight metabolites related to MDD with FR was developed, which provided a predictive accuracy, sensitivity, and specificity of nearly 0.85 for discrimination of MDD patients with FR from HCs. Metabolomic signatures might exist long-term in MDD patients and could have a persisting impact on physical health, despite FR of the disease.

## Supplementary Information

Supplementary Information.

## Data Availability

The datasets generated during and/or analysed during the current study are available from the corresponding author on reasonable request.
