# Supplementary material for: Metabolomics-based discrimination of patients with remitted depression from healthy controls using 1H-NMR spectroscopy
Source: Sci Rep. 2021 Aug 2;11:15608. doi: 10.1038/s41598-021-95221-1 (PMC8329159; doi:10.1038/s41598-021-95221-1)
Supplement: Supplementary file 1 — Supplementary Information. [file 41598_2021_95221_MOESM1_ESM.docx]

**Supplementary information**

**Metabolomics-based discrimination of patients with remitted depression from healthy controls using ^1^H-NMR spectroscopy**

Ching-I Hung, Gigin Lin, Meng-Han Chiang, Chih-Yung Chiu*


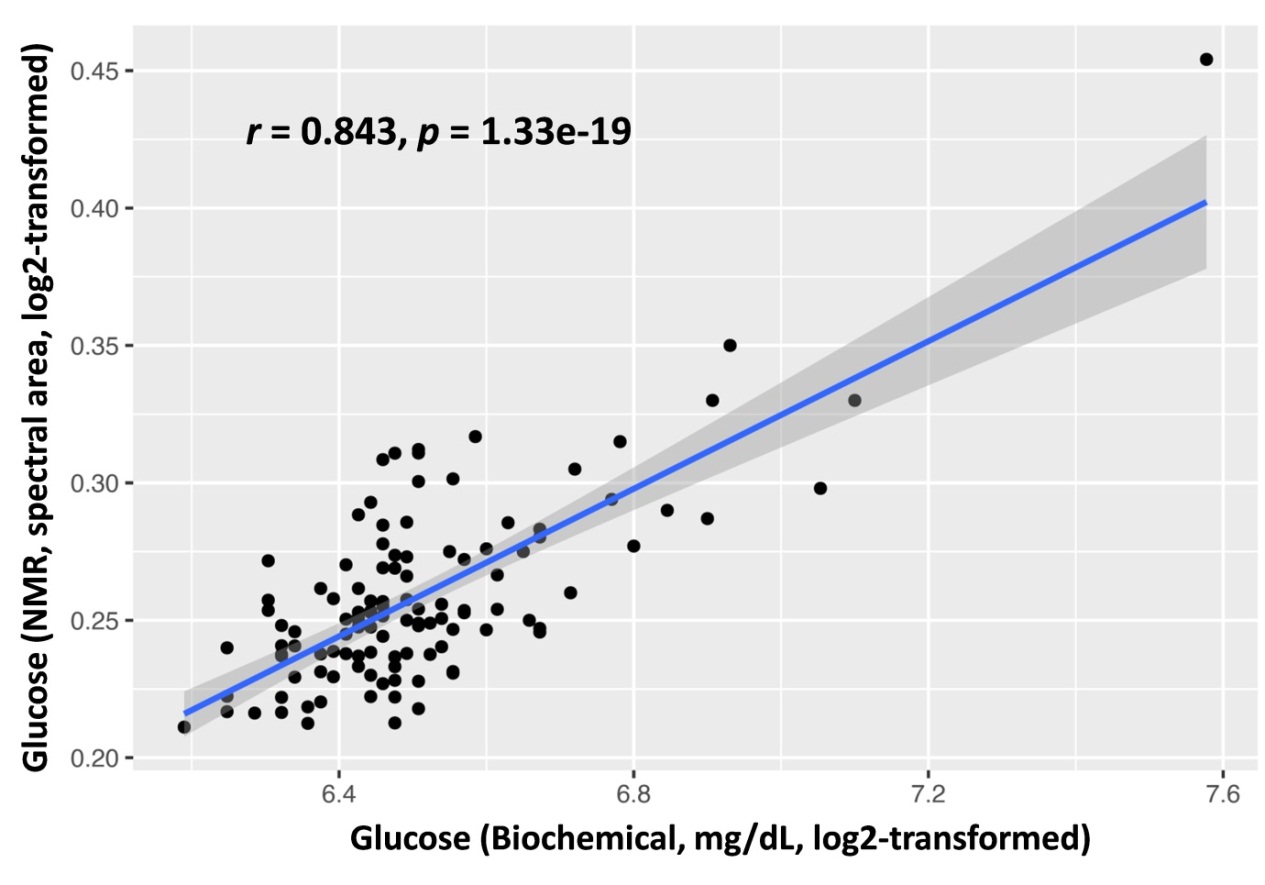


**Supplementary Figure S1. Correlation of glucose between plasma biochemical concentration and NMR spectral area.** The plasma glucose concentrations from a biochemical analysis were significantly correlated with the glucose found in the NMR-spectra (*r* = 0.843, *p* = 1.33e-19). The x- and y-axes were both log2-transformed.

**Supplementary Table S1. ^1^H-NMR assignment results of the identified metabolites in plasma samples.**

| Metabolites | Chemical shift, ppm (multiplicity) |
| --- | --- |
| 3-Hydroxybutyric acid | 2.370-2.391 (d) |
| 3-Hydroxyisobutyric acid | 1.050-1.077 (d) |
| Acetic acid | 1.907-1.914 (s) |
| Acetoacetic acid | 2.270-2.277 (s) |
| Acetone | 2.221-2.224 (s) |
| Alanine | 1.455-1.490 (d) |
| Citric acid | 2.519-2.527 (d) |
| Creatine | 3.918-3.926 (s) |
| Creatinine | 3.034-3.040 (s) |
| Glucose | 5.212-5.245 (d) |
| Glutamine | 2.403-2.409 (m) |
| Glycine | 3.548-3.565 (s) |
| Guanidoacetatic acid | 3.683-3.800 (s) |
| Histidine | 7.760-7.783 (s) |
| Isoleucine | 0.910-0.936 (d) |
| Lactic acid | 4.083-4.125 (d) |
| Leucine | 0.947-0.968 (t) |
| Lysine | 1.490-1.526 (m) |
| Methanol | 3.352-3.360 (s) |
| N,N-Dimethylglycine | 2.868-2.935(s) |
| Phenylalanine | 7.301-7.448 (m) |
| Proline | 2.322-2.357 (m) |
| Propylene glycol | 1.123-1.148 (d) |
| Pyruvic acid | 2.357-2.369 (s) |
| Succinic acid | 2.394-2.397 (s) |
| Tyrosine | 6.876-6.908 (m) |
| Valine | 1.017-1.050 (d) |

VIP, Variable Importance in Projection; ppm, parts per million; d, doublet; s, singlet; t, triplet; m, multiplet.
